# The Applied Weighted Slide Metric (AWSM) Tool: Creation of a Standard Slide Design Rubric

**DOI:** 10.30476/JAMP.2021.91010.1428

**Published:** 2022-04

**Authors:** GABRIEL SUDARIO, SHANNON TOOHEY, WARREN WIECHMANN, JON SMART, MEGAN BOYSEN-OSBORN, JULIE YOUM, SOPHIA SPANN, ALISA WRAY

**Affiliations:** 1 Department of Emergency Medicine, University of California, Irvine, School of Medicine, California, USA

**Keywords:** Instructional, Medical education, Creation

## Abstract

**Introduction:**

Lectures are a standard aspect across all realms of medical education. Previous studies have shown that visual design of presentation slides can affect learner outcomes. The purpose of this study was to develop a slide design rubric grounded in evidence-based, multimedia principles to enable objective evaluation of slide design.

**Method:**

Using the principles described in Mayers’ Principles of Multimedia Learning and Duarte’s Slide:ology, the authors extracted nineteen items important for slide design. We developed an online, rank-item, survey tool to identify the importance of each item among medical educators. Respondents selected which slide design principles they felt were important when attending a lecture/didactic session and ranked their relative importance.

**Results:**

We received 225 responses to the survey. When asked to specifically rank elements from most important to least important, participants gave the most weight to “readability of figures and data” and “[lack of] busy-ness of slide.” The lowest ranked elements were “transitions and animations” and “color schemes”. Using the results of the survey, including the free response, we developed a rubric with relative weighting that followed our survey data.

**Conclusion:**

With this information we have applied values to the various aspects of the rubric for a total score of 100. We hope that this rubric can be used for self-assessment or to evaluate and improve slides for educators. Future research will be focused on implementing and validating the slide design survey and ensuring it is easily usable with a high inter-rater reliability and whether self-assessment with the rubric improves presentation design and education quality.

## Introduction

Lectures are traditionally a large part of undergraduate, graduate and continuing medical education. Throughout medical school, the average student spends thousands of hours participating in lectures, which typically rely on presentation slides ( 1 ). There are many “best practices” for slide preparation from business, design and education literature that provide guidance on slide background, font size, color choice, headings, text and images ( 2 , 3 ). 

The visual design of presentation slides may affect learner outcomes ( 4 ). Mayer and colleagues propose two assumptions in multimedia learning: humans process pictorial and verbal material through separate systems (dual channel assumption) and each channel is limited in the volume of material that can be processed (limited capacity assumption) ( 5 ). The cognitive theory of multimedia learning suggests that our memory is present in three stores: sensory, working and long term memory. These stores work together as an active processing system, with inherent limits to the working memory store. Thus, a key learning tenet is to reduce the demands of cognitive processing through effective multimedia design ( 4 ). 

The steps of selecting, organizing, and integrating require increasing amounts of cognitive processing, and as there is a limited or fixed capacity, any additional burdens can disrupt learning. In other words, if one type of processing is increased, then another type has to be decreased to compensate. *Extraneous processing* is cognitive processing that does not support the instructional goal and is caused by poor design of the multimedia presentation. Minimizing extraneous processing will ensure that more cognitive capacity is available for essential processing. Excess extraneous processing results in decreased learning. *Essential processing* is the cognitive processing required to select words/images and represent them in working memory. The more complex the material, the more essential processing required, thereby limiting the amount of cognitive capacity available for generative processing. Increased essential processing results in rote learning, which is characterized by good retention, but poor transfer. *Generative processing* is the cognitive processing required to make sense of the material, accomplished by organizing material and integrating it with prior knowledge ( 6 ).

In summarizing the key research in multimedia principles that decrease cognitive load (coherence, signaling, redundancy, spatial contiguity and temporal contiguity), Mayer and Fiorella found educationally significant median effect size on problem-solving transfer tests when these design principles were applied to learners in the experimental setting ( 4 ). This suggests that better design has a positive effect on learning. Mayer principles of multimedia design that demonstrated statistically significant positive effects are summarized in Table 1. 

**Table 1:** Summary of Mayer’s Design Principles with a positive effect size

Principle	Conditions in which they improve learning	Median Effect Size
*Theoretical Rationale for effectiveness:* Dual channel assumption of the Cognitive Theory of Multimedia Learning (CTML)		
Multimedia	When instruction is presented in words and pictures compared to words alone	*d* = 1.35
*Theoretical Rationale for effectiveness:* Reduces extraneous processing		
Coherence	When the following are eliminated: interesting but irrelevant words, unneeded words and symbols, interesting but irrelevant music	*d* = 0.86
Signaling	When cues highlight important information	*d* = 0.70
Redundancy	When graphics and narration do not also include printed or onscreen text	*d* = 0.72
Spatial Contiguity	When corresponding words and images are presented near one another (integrated), instead of separated	*d* = 0.82
Temporal Contiguity	When corresponding words and images are presented simultaneously, instead of successively	d = 1.31
*Theoretical Rationale for effectiveness:* Manages demand of essential processing		
Segmenting	When complex material is broken up into smaller parts and learners control the pace of instruction	*d* = 0.67
Pre-training	When lessons are preceded with names and characteristics of key information	*d* = 0.78
Modality	When instruction is presented using pictures and spoken words instead of pictures and printed or on-screen words	*d* = 1.00
*Theoretical Rationale for effectiveness:* Supports generative processing		
Personalization	When speech is presented in a conversational style	*d* = 1.00
Voice	When speech is presented in a dynamic human voice	*d* = 0.74
Image	When a static image of the presenter is not included in the lesson	*d* = 0.20
Embodiment	When lessons are presented by instructors or agents that use human-like gestures and movements	*d* = 0.58
Generative Activity	When lessons include prompts to engage in active learning	*d* = 0.71

It is important to highlight that most of the empirical data on multimedia design, specifically by Mayer ( 6 ), are based on experimental studies using produced multimedia lessons. These are lessons created for the purpose of the study, administered in a study setting, and often contained visual images and spoken word. The literature on captured multimedia lessons – lessons given in real, non-experimental classroom settings – is not as robust, though some studies are applicable here. 

**Figure 1:**
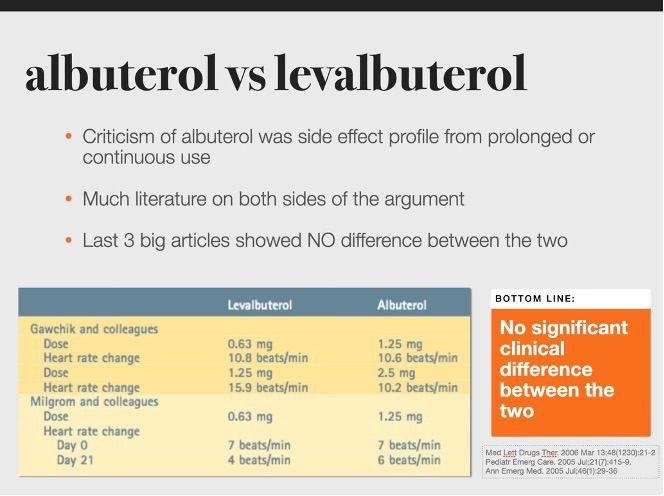
Example of slide with good assertion-evidence structure and multimedia design principles

According to Strauss et al., several design techniques may minimize cognitive overload during slide presentations: text emphasis, relevant visuals, full sentence titles, and minimizing the volume of text/bullet points ( 7 ). Inoue-Smith and colleagues showed that college students had higher preference for slides that chose a solid color background; had limited content, avoided long sentences, did not use all capital letters, and included images and graphics ( 8 ). Garner and Alley found that students had better comprehension and retention from slides that adhere to an assertion-evidence structure, which incorporates six principles of multimedia learning (multiple representation, contiguity, redundancy, modality, coherence and signaling). Slides that abide by assertion-evidence slide structure display a succinct text headline (assertion) with graphic evidence that supports or explains the assertion ( 9 ). Figure 1 displays some of these principles including spatial contiguity (relevant on-screen text is in close physical proximity to the related images or tables), coherence (only relevant information present) and signaling (topic and take-away point from the slide is highlighted and differentiated). 

Faculty development may be used to educate instructors on sound principles for slide design. A rubric may be an important tool to assist faculty in recalling these concepts. Hung, et al. crafted a design rubric as a formative assessment tool for English learners’ presentation slides. This rubric asked the following questions related to visual design:

- Did the author adopt a visual theme?
- Did the author carefully design the use of color and typology to reﬂect the selected visual theme?
- If chosen to use, did the author make meaningful use of available visual elements, such as graphics, to construct meaning in a cohesive manner?
- How did the visual design represented in the multimodal text enable or limit the author’s communication of meaning?

They were able to show that students who received formative feedback from the rubric had significant improvement in presentation performance ( 10 ). However, in our opinion, many of Hung’s slide rubric questions were subjective, not grounded in multimedia theory and may not be useful as a rapid evaluation method of slide design. We believe that a more objective, granular and comprehensive evaluation tool may allow for frequent, useful feedback for improving presentation performance.

The purpose of this study was to develop a rubric that addresses multiple aspects of slide design, grounded in evidence-based, multimedia principles. Such a rubric, whether used as an evaluation tool or best practice checklist, makes these multimedia principles accessible to educators and enables objective evaluation of slide design. The literature demonstrates that multimedia design, and therefore slide design, has a statistically significant positive impact on learning ( 10 ). We feel that it is imperative that instructors leverage this empirical data to help their learners learn. 

## Methods

### Selection of Multimedia Principles

**Figure 2:**
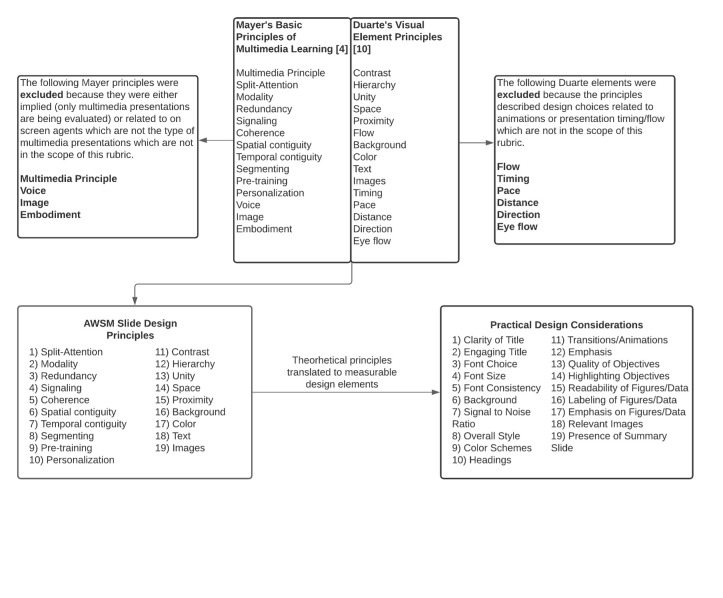
Selection of slide design principles

An expert panel of seven medical educators with extensive experience in undergraduate and graduate medical education, educational fellowship leadership and with advanced degrees in education came together to select rubric criteria. In four hours of panels meetings taking place over one month time, our panel analyzed and identified 29 multimedia principles presented in Mayer’s Basic Principles of Multimedia Learning and Duarte’s Slide:ology ( 4 , 11 ). Of the 14 basic multimedia principles established by Mayer, four were excluded. The multimedia principle, which states that words and pictures are more effective than words alone, was excluded as our rubric was only being used to evaluate multimedia presentations. Voice, image and embodiment principles are specific to the use of on-screen agents in presentations without live speakers and were also excluded from the final list of guiding principles ( 4 ). Duarte organizes her slide design elements into three subcategories of arrangement (contrast, hierarchy, unity, space, proximity, flow), visual elements (background, color, text, images) and movement (timing, pace, distance, direction, eye flow). Because the principles of flow, timing and pace were related to the speaker’s words, they were excluded. Principles of eye flow, distance and direction were excluded as they are design choices directly related to the effectiveness of slide animations which was outside of the scope of this work. Using the final 19 principles, we created an aligned list of practical implementations of the multimedia theory that could be evaluated by an observer. Each of these practical design principles aligns with one or more multimedia principle (see Figure 2). 

### Survey Development

We developed an online survey tool to identify the importance of each of the above items among medical educators. Respondents selected which slide design principles they felt were important when attending a lecture/didactic session and ranked their relative importance. Respondents were able to select as many items as they wished. The survey also collected basic demographic data of the respondents, including current role, age and frequency of lectures given to learners. The process to select the slide design principles helped to establish the content validity for this survey. The survey was then piloted with a team of medical education experts to establish face validity by using the survey to rate a sample of slide presentations from medical educators. The team independently rated slide designs using the survey and the results were compared and discussed. Questions were revised based on discussion and consensus. Prior to deploying the survey, we piloted it with a small sample of faculty members to ensure the clarity of its questions.  

### Recruitment

We sent the survey to a national convenience sample of undergraduate and graduate medical educators through various listservs. These listservs included DR-ED (an electronic discussion group for medical educators) ( 12 ), the Director of Clinical Skills (DOCS) group and the emergency medicine Council of Residency Directors (CORD). Based on enrollment in these listservs, we estimate that these groups collectively represent a sample size of 5500 medical educators working in both undergraduate and graduate medical education in the United States. We selected these groups as we felt they represented educators who frequently give and receive medical lectures and find value in high quality slide design. 

### Ranked Weights

Using Microsoft Excel, the ranked weight of each item was calculated using the average ranking for each answer choice. We applied weights in reverse, such that the respondent’s most preferred choice had the largest weight ( 11 ) and decreased by 1 for each decrease in order of importance. To avoid overrepresenting the importance of an item we did not exclude non-respondents (i.e., for respondents who did not select a particular item in the first place, that item received a score of 0 for that respondent). 

### Ethical Consideration

This study qualified as exempt by the University of California, Irvine Institutional Review Board.

## Results

### Respondent Statistics

We received 225 responses to the survey (4% response rate). The demographics for respondents are shown in Table 2. Survey respondents were usually an attending level physician, in the 35-44 age range, who gave presentations/lectures on an at least monthly basis. 

**Table 2:** Survey respondent demographic information

	Demographic	Frequency	Percentage
Title	Attending Physician	116	51.56%
	Resident Physician	7	3.11%
	Advanced Practitioner	2	0.89%
	Other	100	44.44%)
Age	18-24	0	0.00%
	25-34	23	10.22%
	35-44	68	30.22%
	45-54	62	27.56%
	55-64	46	20.44%
	65+	26	11.56%
Frequency of giving lectures	Rarely	20	9.83%
	Yearly	40	17.86%
	Monthly	115	51.34%
	Weekly or more	49	21.88%

### Outcomes

Respondents initially marked the slide elements they considered important in a binary yes/no fashion. A summary of design element frequency and weighted rank is summarized in Table 3. The most commonly selected elements were “readability of figures and data” (n=182, 80.89%) and “ busy-ness of slides” (n=172, 76.44%). These were closely followed by “use of relevant images” (n=161, 71.56%), “appropriate emphasis on major points” (n=158, 70.22%), and “appropriate emphasis of important points in figures and data” (n=158, 70.22%).

**Table 3:** Slide design elements frequency and weights

Design Element	Frequency	Percentage	Ranked Weight
Readability of figures and data	182	80.89%	15.43
Busy-ness of slide	172	76.44%	15.45
Use of relevant images	161	71.56%	12.58
Appropriate emphasis of important points in figures/data	158	70.22%	12.48
Appropriate emphasis on major points	158	70.22%	14.49
Font size	147	65.33%	11.8
Font/background contrast	135	60.00%	10.64
Presence of summary slide	116	51.56%	8.36
Quality of objectives	116	51.56%	9.89
Highlighting objectives throughout lecture	104	46.22%	8.27
Clarity of title	91	40.44%	6.15
Font consistency	89	39.56%	5.64
Overall lecture style/theme	77	34.22%	5.78
Font choice	71	31.56%	3.99
Engaging or interesting title	70	31.11%	4.45
Labeling presence and consistency in figures/data	68	30.22%	4.16
Headings and subheadings	56	24.89%	3.61
Color schemes	53	23.56%	2.8
Transitions and animations	42	18.67%	1.87

Conversely, the elements that participants selected less frequently were “transitions and animations” (n=42, 18.67%) and “color schemes” (n=53, 23.56%). Other elements selected less often were “headings and subheadings” (n=56, 24.89%), “labeling presences and consistency in figures and data” (n=68, 30.22%), and “engaging or interesting title” (31.11%).

When asked to specifically rank elements from most important to least important, participants gave the most weight to “readability of figures and data” and “ busy-ness of slide”, as shown in Table 3. The lowest ranked elements were “transitions and animations” and “color schemes”.  

Participants were also given the opportunity to leave a free response to other factors that were not covered in the survey. Of the participants who left free response comments (n=79, 33%), 20.24% (n=46) noted that minimizing words was an important factor and 13.9% (n=31) mentioned simplicity. Finally, 10.21% (n=23) of those leaving comments mentioned the importance of coherence between spoken words and material visualized on the slides. 

Using the results of the survey, including the free response, we developed a rubric with relative weighting that followed our survey data. We initially applied the weighted values for each principle to the rubric (rounded to the nearest whole number). However, we found this rubric totaled 156 points which is excessively large for a rubric. Additionally, we realized that while the weighted averages gave us a clear order and relative importance of the principles, the actual values for each rubric did not need to correspond to the survey, just the relative point values.  For example, based on the survey use of relevant images valued 12.58 and clarity of title valued 6.15. We used the initial weights as a starting point. Our expert panel of educators then reviewed each principle and decreased the point values in the rubric by approximately 50% to make the overall total 100 while maintaining the relative importance of each item. To continue the example above, relevant images was adjusted to be worth 8 points, while clarity of title was adjusted to be worth 4 points.

## Discussion

Overall, respondents rated elements of slide design favoring minimalism, such as busy-ness of slide and appropriate emphasis on key information, as most important. This aligns well with many of Mayer’s multimedia principles including the split-attention principle, redundancy principle, the signaling principle and other techniques to minimize extraneous processing (coherence, redundancy, and spatial contiguity) ( 4 ) While best practices from the business literature ( 2 ) and Duarte ( 11 ) focus on stylistic choices such as color schemes transitions and font, our results suggest that these aspects of slide design are less important. This is not surprising and likely due to the fact that medical educators weigh content over style. 

While Mayer and Duarte created a framework and best practices guidelines for multimedia and slide design, our literature review did not find any previous studies that attempt to prioritize these components. Our findings also suggest that medical educators prefer to focus on components of slide design that are directly related to evidence-based multimedia principles, while more stylistic characteristics were deemed less important. This would imply that further faculty development and training in slide design should emphasize the importance of Mayer’s principle in designing slides. This should include the basics in ensuring readability, relevance and emphasis when designing multimedia presentations.  While many novice educators may be overwhelmed with the idea of improving slide “design,” our tool can be used as an evidenced based checklist and takes no formal training in visual design to implement. 

Our survey data also shows there seems to be a correlation between the frequency of design elements selected as “important” and the relative rank of that item. Because high frequency items are also highly ranked, this could indicate that our survey was reliable in identifying important factors in slide design. 

Free responses from the survey offered support for these conclusions, i.e., common themes focused on “busy-ness”, number of words on slides and presentations. Survey respondents most commonly mentioned the importance of decreasing slide busy-ness, decreasing words and bullet points on slides and the idea of simplicity, which we felt was already included as an element in the survey. Such responses may suggest that medical educators value decreasing busy-ness so heavily that even though it was included as an item for ranking they wished to emphasize its importance by including in free responses as well.

### Limitations

One limitation of our study was utilizing the listservs to collect responses, as they represent a small sampling of medical educators in the US. Additionally, while DR-ED and DOCS have representation from the majority of the accredited medical schools in the US, the survey was not sent to other graduate medical education listservs and there is the possibility that other graduate medical education specialties may have responded differently. We recognize the small response rate within our chosen sample which is attributed to the nature of soliciting survey responses through listserv platforms. Another limitation is the possibility of a self-selection bias, where only those that are interested in slide design may have completed the survey. However, we feel that this bias would have benefited the study as those who find slide design more important are more likely to have strong opinions about slide design and knowledge of best practices. Overall, we understand that this specialized tool was developed from the input of a narrow range of users which may affect the ability to generalize it to larger medical education settings.

There are also some limitations of the final development of the rubric. While we attempted to utilize large expert opinion to weight each item of the rubric it is still not definitive that the weights are an accurate representation of the quality of the slide design. However, we believe it is the best attempt to accurately measure each item’s value and there are no other rubrics that attempt to measure various aspects of slide design to this degree of detail. We believe it is an excellent starting point based on previous literature, established principles, and expert opinion. We believe it can be further calibrated with utilization and future research.

We also recognize the limitations inherent in reviewing only the slides instead of the live or recorded lecture presentation. In our slide review, it was not possible to distinguish static images from dynamic animations. The progression of steps in an animation do have some benefits from the temporal contiguity and segmenting principles that we could not capture or analyze. Additionally, pointing for emphasis may have occurred by hand, laser pointer, or even mouse, which all are beneficial based on the signaling principle – these activities are not represented on the slides reviewed and therefore could not be captured or analyzed.

## Conclusions

Our goal was to create a slide design rubric that assesses the quality of slide decks for medical educators. While the initial components were selected by an expert panel of medical educators based on established multimedia principles and expert opinion, we developed the survey to collect further information from the medical educator community to ensure various aspects of the rubric were appropriately weighted. With this information we have applied values to the various aspects of the rubric for a total score of 100. This study reports the initial results of that data. 

We hope that this rubric can be used for self-assessment or to evaluate and improve slides for educators. Future research will be focused on implementing and validating the slide design survey and ensuring it is easily usable with a high inter-rater reliability. Specifically, we plan to implement our rubric by targeting lectures given at a national medical education conference, establishing various reliability and validity measures. Longer term studies will evaluate whether self-assessment with the rubric improves presentation content and design and improves education quality. 

## Acknowledgement

All authors contributed equally to design, data collection, writing and editing of the manuscript. No financial disclosures or sponsors. 

**Conflict of Interest:** None declared.
